# Dynamic Increase in Extracellular ATP Accelerates Photoreceptor Cell Apoptosis via Ligation of P2RX7 in Subretinal Hemorrhage

**DOI:** 10.1371/journal.pone.0053338

**Published:** 2013-01-08

**Authors:** Shoji Notomi, Toshio Hisatomi, Yusuke Murakami, Hiroto Terasaki, Shozo Sonoda, Ryo Asato, Atsunobu Takeda, Yasuhiro Ikeda, Hiroshi Enaida, Taiji Sakamoto, Tatsuro Ishibashi

**Affiliations:** 1 Department of Ophthalmology, Graduate School of Medical Sciences, Kyushu University, Fukuoka, Japan; 2 Clinical Research Institute, National Hospital Organization Kyushu Medical Center, Fukuoka, Japan; 3 Department of Ophthalmology, Graduate School of Medical Sciences, Kagoshima University, Kagoshima, Japan; Center for Regenerative Therapies Dresden, Germany

## Abstract

Photoreceptor degeneration is the most critical cause of visual impairment in age-related macular degeneration (AMD). In neovascular form of AMD, severe photoreceptor loss develops with subretinal hemorrhage due to choroidal neovascularization (CNV), growth of abnormal blood vessels from choroidal circulation. However, the detailed mechanisms of this process remain elusive. Here we demonstrate that neovascular AMD with subretinal hemorrhage accompanies a significant increase in extracellular ATP, and that extracellular ATP initiates neurodegenerative processes through specific ligation of Purinergic receptor P2X, ligand-gated ion channel, 7 (P2RX7; P2X7 receptor). Increased extracellular ATP levels were found in the vitreous samples of AMD patients with subretinal hemorrhage compared to control vitreous samples. Extravascular blood induced a massive release of ATP and photoreceptor cell apoptosis in co-culture with primary retinal cells. Photoreceptor cell apoptosis accompanied mitochondrial apoptotic pathways, namely activation of caspase-9 and translocation of apoptosis-inducing factor (AIF) from mitochondria to nuclei, as well as TUNEL-detectable DNA fragmentation. These hallmarks of photoreceptor cell apoptosis were prevented by brilliant blue G (BBG), a selective P2RX7 antagonist, which is an approved adjuvant in ocular surgery. Finally, in a mouse model of subretinal hemorrhage, photoreceptor cells degenerated through BBG-inhibitable apoptosis, suggesting that ligation of P2RX7 by extracellular ATP may accelerate photoreceptor cell apoptosis in AMD with subretinal hemorrhage. Our results indicate a novel mechanism that could involve neuronal cell death not only in AMD but also in hemorrhagic disorders in the CNS and encourage the potential application of BBG as a neuroprotective therapy.

## Introduction

Age-related macular degeneration (AMD) is the leading cause of irreversible vision loss in the elderly in the developed world [1]. Among Americans, the estimated prevalence of AMD is projected to increase by more than 50% by the year 2020 [2]. In the neovascular form of this disorder, severe visual loss commonly occurs as a result of the invasion of abnormal blood vessels from the choroidal circulation, namely choroidal neovascularization (CNV), which induces irreversible damage to the overlying retina [3]. CNV could be induced by focally increased inflammatory and proangiogenic factors and/or by a decrease in anti-angiogenic factors. Various clinical as well as experimental studies have shown that vascular endothelial growth factor (VEGF), a proangiogenic glycoprotein, could be the most important factor for development of CNV [4]. In recent years, pharmacological inhibition of VEGF has offered the first opportunity to improve visual outcomes in patients diagnosed with this disorder [5]. Intraocular injections of an anti-VEGF antibody, such as ranibizumab or bevacizumab, have improved visual outcomes in several clinical trials [6]. However, patients with predominant subretinal hemorrhage, a commonly encountered event in neovascular AMD, still have poor visual prognoses [7]. Pneumatic displacement or surgical evacuation of subretinal blood with the use of recombinant tissue plasminogen activator (tPA) failed to improve the visual outcomes of patients with submacular hemorrhage due to AMD in a controlled clinical trial [8].

The most crucial stage of severe visual impairment is photoreceptor loss due to development of the CNV and related events such as subretinal hemorrhage or exudative retinal detachment in neovascular AMD [3]. Several clinical and experimental studies have shown that subretinal hemorrhage induces severe photoreceptor cell apoptosis [9]–[10] in line with severe tissue damage due to subarachnoid or intracerebral hematoma in the central nervous system (CNS) [11]–[12]. Previous studies have found that several potential neurotoxic agents were released from extravascular blood, such as hemoglobin [13], iron [10] [14]–[15], or glutamate [16], indicating that neurotoxic agents released from extravascular blood can be potential therapeutic targets.

Photoreceptor degeneration involves the activation of several signaling pathways of regulated cell death that can constitute potential therapeutic targets. Accordingly, attempts have been made to inhibit caspases, which play major roles in the apoptotic machinery [17]–[18], although pharmacological pan-caspase inhibitors largely failed to preserve the structures and functions of photoreceptors [19]–[20]. Caspases can be activated as a result of mitochondrial outer membrane permeabilization (MOMP) and the subsequent mitochondrial release of cytochrome *c* that triggers the Apaf-1 (apoptotic protease activating factor 1) apoptosome activation. MOMP also results in the mitochondrial release of apoptosis-inducing factor (AIF), which translocates to the nucleus and participates in the caspase-independent peripheral chromatin condensation and large-scale DNA fragmentation [21]. These findings suggest the existence of redundant cell death mechanisms downstream of MOMP [19] [22]–[23].

An alternative strategy to inhibit the mitochondrial apoptotic pathway is to intercept the initiating upstream proapoptotic signals. Recently, adenosine-5′-triphosphate (ATP) has been discovered as a major extracellular messenger that can contribute to lethal signaling [24]. Extracellular ATP can act on purinergic receptors, which are classified into two classes, the ionotropic, ligand-gated P2X receptors and the metabotropic, G protein-coupled P2Y receptors [25]. Among the seven subtypes of mammalian P2X receptors [26], the P2X7 receptor (P2RX7) differs from other P2X receptor subtypes by its long cytoplasmic, carboxy-terminal tail (240 amino acids) and mediates cellular signals that can trigger cell death [27]–[28]. P2RX7 is widely expressed in various organs, including those of immune system [29] and central nervous system [30]. In the retina, P2RX7 is expressed in Müller glia [31] as well as in both inner and outer retinal neurons, including retinal ganglion cells [32] and photoreceptor cells [33]–[34]. Furthermore, Fletcher and her group reported that P2RX7 has physiological functions as a neurotransmitter receptor in the retina [35], while photoreceptor cells have been shown to undergo apoptosis in response to an excess of extracellular ATP [36]. Recently, we have shown that photoreceptor cell apoptosis involves P2RX7 activation with caspase-8, -9 cleavage and mitochondrio-nuclear translocation of AIF. Moreover, photoreceptor cell apoptosis can be attenuated by Brilliant Blue G (BBG), a pharmacological P2RX7 antagonist that acts by blocking the interaction between extracellular ATP and P2RX7 [37]. Indeed, BBG administration can confer neuroprotective effects in several models of Alzheimer’s, Parkinson’s disease, and spinal cord injury [38]–[40] as well as in the retina [41]–[42]. BBG is also known as an adjuvant approved for intraoperative use in ocular surgery. In chromovitrectomy, vital dyes are introduced to improve the visualization of intraocular tissues for specific procedures, such as internal limiting membrane (ILM) peeling during vitrectomy [43]–[44].

Hence, we hypothesized that ATP acting on P2RX7 is involved in the pathogenesis of photoreceptor loss in subretinal hemorrhage. To examine the clinical relevance of this hypothesis, we compared extracellular ATP concentrations in human vitreous samples from patients with retinal diseases, including AMD. Our results showed that increased extracellular ATP substantially contributes to photoreceptor loss and that BBG provides substantial neuroprotective effects in cases of subretinal hemorrhage.

## Materials and Methods

### Ethics Statement

Procedures using human samples were conducted in accordance with the Declaration of Helsinki and approved by the Kyushu University Institutional Review Board for Clinical Research. We obtained written informed consent from all the participants. All procedures using mice were performed in strict accordance with the guidelines for Association for Research in Vision and Ophthalmology (ARVO) and Proper Conduct of Animal Experiments (Science Council of Japan). The experimental protocol was approved by the Animal Care and Use Committee of Kyushu University. All efforts were made to minimize the number of animals used and their suffering.

### Animals

Adult (8 weeks of age) male C57BL6JJcl mice (CLEA, Tokyo, Japan) and isogenic P2rx7^−/−^ mice, the latter kindly provided by Pfizer Inc. (Gorton, CT), were used in this study. Recently, the rd8 (retinal degeneration 8) mutation was reported to be widely inherited across strains supplied by common commercial vendors and ES cell lines [45]–[46]. We confirmed that P2rx7^−/−^ mice do not carry the rd8 mutation by DNA sequencing (Supporting Information S1). All procedures using mice were performed in strict accordance with the guidelines for Association for Research in Vision and Ophthalmology (ARVO) and Proper Conduct of Animal Experiments (Science Council of Japan). The experimental protocol was approved by the Animal Care and Use Committee of Kyushu University. All efforts were made to minimize the number of animals used and their suffering.

### Human Vitreous Samples

Procedures using human samples were conducted in accordance with the Declaration of Helsinki and approved by the Kyushu University Institutional Review Board for Clinical Research. We obtained written informed consent from all the participants. Vitreous samples (1.0–1.2 ml) were collected from patients who underwent pars plana vitrectomy at the Vitreoretinal Center in Kyushu University Hospital and Kagoshima University Hospital, for the treatment of macular hole (MH, *n = *10), epiretinal membrane (ERM, *n = *10), or age-related macular degeneration (AMD) with vitreous hemorrhage (VH) (*n = *15). The clinical characteristics of the patients are summarized in Table 1 and Supporting Information S2.

**Table 1:** Clinical characteristics of patients with MH, ERM, and AMD with VH.

Characteristics	MH	ERM	AMD with VH	*P*
*n*	10	10	15	
Age (years)				
Mean (SD)	67.9 (6.8)	70.7 (6.4)	70.7 (10.4)	NS
Gender, *n*				
Male	4	2	9	
Female	6	8	6	NS

The patients underwent pars plana vitrectomy and the collected vitreous samples were subjected to ATP measurement by luciferase assay. There were no significant differences in age or sex ratio among the three groups.

### ATP Measurements

The ATP levels of collected samples (100 µl each in 96-well microplates; BD Falcon, Franklin Lakes, NJ) were immediately determined by using luciferin-luciferase reaction buffer (ATP bioluminescence assay kit, FL-AA; Sigma-Aldrich, St. Louis, MO) and a Flex Station 3 Multi-Mode Microplate Reader (Molecular Devices, Sunnyvale, CA). The ATP levels detected by the luminometer were expressed in relative light units (RLU).

### Mouse Blood Collection

Mice were anesthetized with an intraperitoneal injection of pentobarbital and exsanguinated via cardiac puncture into collection tubes containing heparin. For the isolation of plasma, collected whole blood was immediately centrifuged at 18000 g for 60 seconds at 4°C to isolate plasma as previously described [47]. For the isolation of erythrocytes, the plasma, platelets, and leukocytes were removed as supernatant and buffy coat after centrifugation at 900 g for 3 min. Isolated erythrocytes were resuspended in medium supplemented with 0.5% bovine serum albumin to the corresponding final hematocrit. To determine ATP levels after hemolysis, whole blood or isolated erythrocytes were lysed in distilled water and then assayed using a firefly luciferase assay (FL-AA) as described above.

### Adult Mouse Primary Retinal Cell Cultures

Adult primary retinal cell cultures were prepared as previously described with minor modifications [48] [22]. Primary retinal cells were cultured in 4-well chambers (Nunc; part of Thermo Fisher Scientific, Bremen, Germany) with Neurobasal-A medium (Invitrogen, Carlsbad, CA) containing B27 supplement without antioxidants (NBA/B27AO–; Invitrogen), 1 µg/ml insulin, and 12 µg/ml gentamicin. To determine the number of adherent photoreceptor cells, immunofluorescent staining was performed with a rabbit anti-recoverin antibody (Millipore, Bedford, MA). For blood clot exposure, we utilized a double chamber system [49]. After 50 µl of whole blood was placed on membranes with micropores in the upper chamber (Transwell; Corning Life Sciences, Lowell, MA), 100 µl of culture medium was added to the upper chamber. Primary retinal cell cultures were prepared in the lower chamber and incubated with or without 10 U/ml apyrase (Sigma-Aldrich, St. Louis, MO) for 24 h. The culture medium was collected and centrifuged, and the ATP levels of supernatants were analyzed by the luciferin-luciferase assay. For exogenous ATP administration, 1 mM ATP (Sigma-Aldrich) was added to the culture medium, and the medium were incubated for 24 h with or without 30 min pre-incubation of BBG.

### Viability Assay in Primary Retinal Cell Cultures

To assess the viability of primary retinal cells, we used calcein AM (2 µM) or MitoTracker Orange CMTMRos (200 nM; M7510; Invitrogen), added for 30 min to primary retinal cell cultures. Then, cultured cells were fixed with 4% paraformaldehyde and recoverin-labeled calcein^+^ or CMTMRos^+^ photoreceptor cells were counted in 10 random fields by blinded observers using ImageJ software. Values are given as the means ± SDs of 10 replicate wells.

### Subretinal Injections

Experimental retinal detachment was performed as previously described [19] [22] [48] [50]. Mice were anesthetized with an intraperitoneal injection of pentobarbital, and their pupils were dilated with topical 1% tropicamide and 2.5% phenylephrine hydrochloride. Then, 2 µl of sodium hyaluronate (Opegan-Hi; Santen Pharmaceuticals, Tokyo, Japan) with a mixture of 1 mM ATP or vehicle PBS, was gently injected into the subretinal space with a 30-gauge needle through the sclera posterior to the limbus to produce focally detached retina. The injection of sodium hyaluronate reproducibly generated similar retinal detachments. Successful retinal detachment was confirmed by ophthalmoscopy and generally involved one third of the retina as previously described [51]. To establish a mouse model of subretinal hemorrhage, autologous blood with a mixture of 50 µM BBG or vehicle PBS was immediately injected into the subretinal space as previously described [10]. The height of retinal detachments was measured at the top of the detached retina in cryosections (Supporting Information S3). Subretinal injections were only performed in the right eye of each animal, and 6 eyes were examined in each group. The mice were sacrificed 24 h after treatment. Their eyes were harvested, frozen at nitrogen liquid temperature, and cryosectioned for histochemical examinations.

### TdT-dUTP Terminal Nick-end Labeling (TUNEL)

TUNEL analysis and quantification of TUNEL-positive cells were performed as previously described using the ApopTag Fluorescein In Situ Apoptosis Detection Kit (Millipore) [22]. Nuclei were counter-stained with propidium iodide or Hoechst 33342. The center of the detached retina was photographed, and the TUNEL-positive cells in the outer nuclear layer (ONL) were counted by two blinded observers. The ratios of photoreceptor cell apoptosis were expressed as the percentage of TUNEL-positive nuclei among the total number of nuclei in the section, and the results are presented as the means ± SDs.

### Electron Microscopy

The posterior segments of enucleated mouse eyes were fixed in PBS containing 1% glutaraldehyde and 1% paraformaldehyde, postfixed in veronal acetate buffer osmium tetroxide (2%), dehydrated in ethanol and water, and embedded in Epon. Ultrathin sections were cut from blocks and mounted on copper grids. The specimens were observed with an H-7770 transmission electron microscope (Hitachi, Tokyo, Japan).

### Immunofluorescence

Immunofluorescence was performed with slight modifications to a previous method [22]. Briefly, rabbit anti-AIF (R&D Systems, Minneapolis, MN), anti-mouse cleaved caspase-9 (Cell Signaling Technology, Beverly, MA), and anti-recoverin (Millipore) were used as primary antibodies and incubated at 4°C overnight. Goat anti-rabbit IgGs conjugated to Alexa Fluor 546 or 647 (Invitrogen) were used as secondary antibodies and incubated at room temperature for 1 h.

### Statistical Analysis

Statistical differences between the two groups were analyzed by means of the Mann–Whitney U-test. Multiple group comparison was performed by ANOVA followed by Tukey–Kramer adjustments. The sex ratio in each group was analyzed by Fisher’s exact test. Differences were considered significant at *P*<0.05 (*) and *P*<0.01 (**). All values were expressed as the means ± SDs.

## Results

### Abundant Photoreceptor Cell Apoptosis in a Mouse Model of Subretinal Hemorrhage

**Figure 1:**
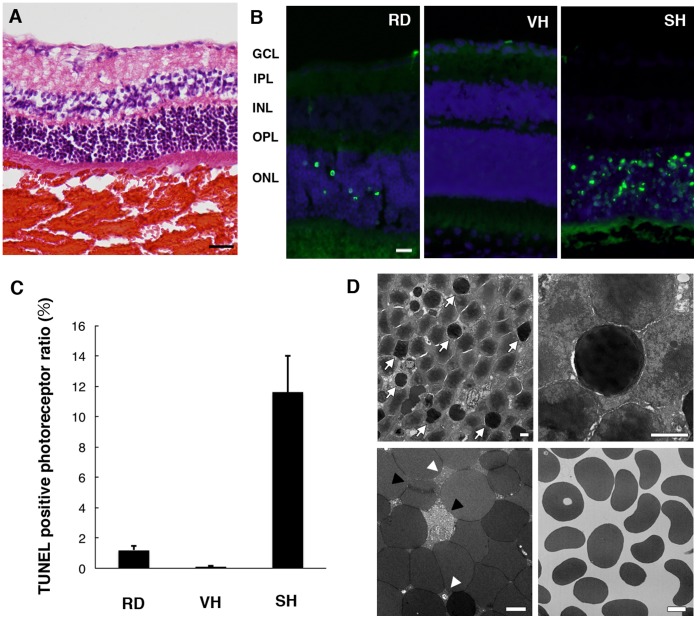
Abundant photoreceptor cell apoptosis in subretinal hemorrhage. Subretinal injection or vitreous injection of autologous blood was performed in C57BL6 mice. (A) A representative image of hematoxylin/eosin staining in subretinal hemorrhage is shown. Scale bar: 100 µm. (B) Some TUNEL^+^ nuclei were detected in the outer nuclear layer (ONL) in retinal detachment (RD), while abundant TUNEL^+^ nuclei were found in subretinal hemorrhage (SH). TUNEL^+^ cells were less detectable in vitreous hemorrhage (VH) (TUNEL in green, propidium iodide in blue). Scale bar: 20 µm. GCL; ganglion cell layer, IPL; inner plexiform layer, INL; inner nuclear layer, OPL; outer plexiform layer, ONL; outer nuclear layer. (C) The numbers of TUNEL^+^ photoreceptors in RD, VH, and SH. (D) Electron microscopy revealed that numerous photoreceptor cells had undergone apoptosis with chromatin condensation (arrows in the top left panel) or cellular shrinkage (top right panel). Some erythrocytes lost their plasma membranes (black arrowheads in the bottom left panel) and hemolytic debris was observed among erythrocytes (white arrowheads in the bottom left panel) in the subretinal hemorrhage. Minimal hemolytic change was observed in the vitreous hemorrhage (bottom right panel). Scale bars: 2 µm.

To investigate the clinically relevant pathology of photoreceptor cell death in neovascular AMD, we created a mouse model of subretinal hemorrhage. Autologous blood drawn from the tail vein was injected into the subretinal space (Figure 1A). Retinal cell death has also been shown to occur after detachment of neurosensory retina [19] [52]. To evaluate the toxicity of subretinal blood to the overlying retina, a control condition was established by performing the retinal detachment using subretinal injections of sodium hyaluronate in line with our previous methods [19]. We examined the height of retinal detachment after injections of sodium hyaluronate or autologous blood and found no significant difference (Supporting Information S3). Abundant TUNEL^+^ photoreceptor cells were detected in the outer nuclear layer (ONL) 24 h after the injections of autologous blood, while quite a small number of apoptotic photoreceptor cells were detected in control retinal detachment within 24 h (Figure 1, B and C). This result indicates that subretinal blood induces massive apoptotic photoreceptor cell death within 24 h and thus would cause irreversible visual impairment in the case of subretinal hemorrhage. Next, to investigate the toxicity of vitreous hemorrhage, autologous blood was injected into the vitreous cavity. Only a few TUNEL^+^ cells were found after this injection in contrast to the case with subretinal hemorrhage (Figure 1, B and C), suggesting that a massive hemorrhage could severely reduce the integrity of photoreceptors, if it were tightly packed into the subretinal space. Moreover, electron microscopy revealed the characteristic signs of photoreceptor cell apoptosis, such as chromatin condensation or cellular shrinkage (Figure 1D). Numerous erythrocytes were observed in the subretinal clots. Some of those erythrocytes lost their plasma membranes with hemolytic debris 24 h after the injections, while minimal hemolytic change was observed in vitreous hemorrhage (Figure 1D). These observations suggest that potential neurotoxic factors were released from the blood clots during erythrocyte hemolysis.

### Increased ATP Levels in the Vitreous of Patients with Age-related Macular Degeneration

**Figure 2:**
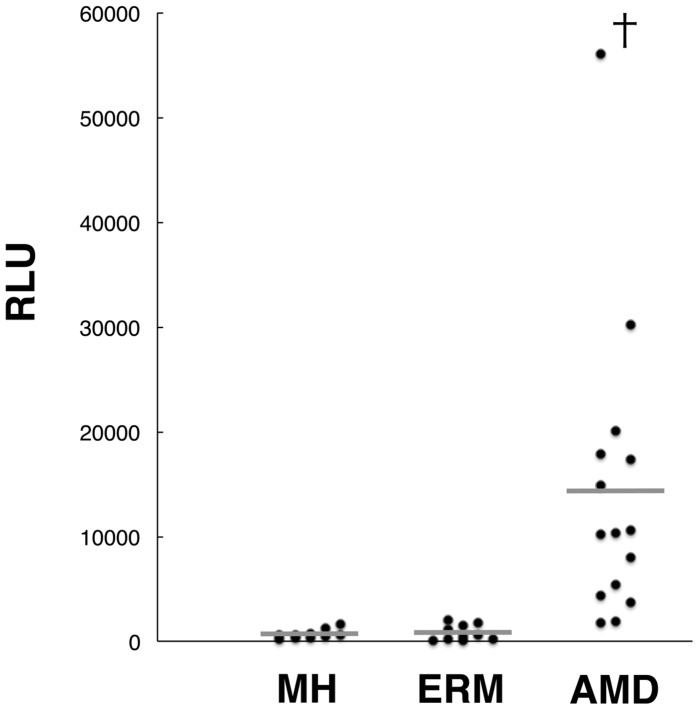
The ATP levels in human vitreous of retinal diseases. Human vitreous samples were collected during vitreoretinal surgery from patients with MH (*n = *10), ERM (*n* = 10), and AMD (*n* = 15). The ATP levels of vitreous samples were determined by luciferin-luciferase assay (RLU: relative light units). †*P*<0.01.

To examine the potential role of extracellular ATP in subretinal hemorrhage, we investigated the intraocular concentrations of ATP by using vitreous samples collected during vitreoretinal surgery from patients with MH, ERM, or AMD with vitreous hemorrhage (VH). There is minimal breakdown of the blood retinal barrier (BRB)–i.e., exudation or bleeding–in MH or ERM. In contrast, vitreous hemorrhage–that is, diffusion of a large amount of blood into the vitreous cavity from the subretinal hemorrhage–often occurs in AMD, and in some of these cases vitrectomy is performed for removal of the vitreous opacity [53]–[54]. ATP levels of vitreous samples were analyzed by luciferin-luciferase assay, which is used to quantify chemiluminescence upon ATP-dependent oxidation of luciferin. Notably, ATP concentrations were much higher in AMD with VH compared to MH or ERM (Figure 2). These results suggest that ATP levels in the subretinal space could be potentially higher than those detected in the vitreous, because extracellular ATP diffuses into the vitreous cavity from the subretinal space. Hence, the local concentration of ATP around photoreceptors might be higher than the concentration of ATP observed in vitreous samples. Moreover, the increase in extracellular ATP may be neurotoxic to the overlying retina in subretinal hemorrhage.

### Extracellular ATP Induces Neurotoxicity in Primary Retinal Cell Cultures with Blood Clot

**Figure 3:**
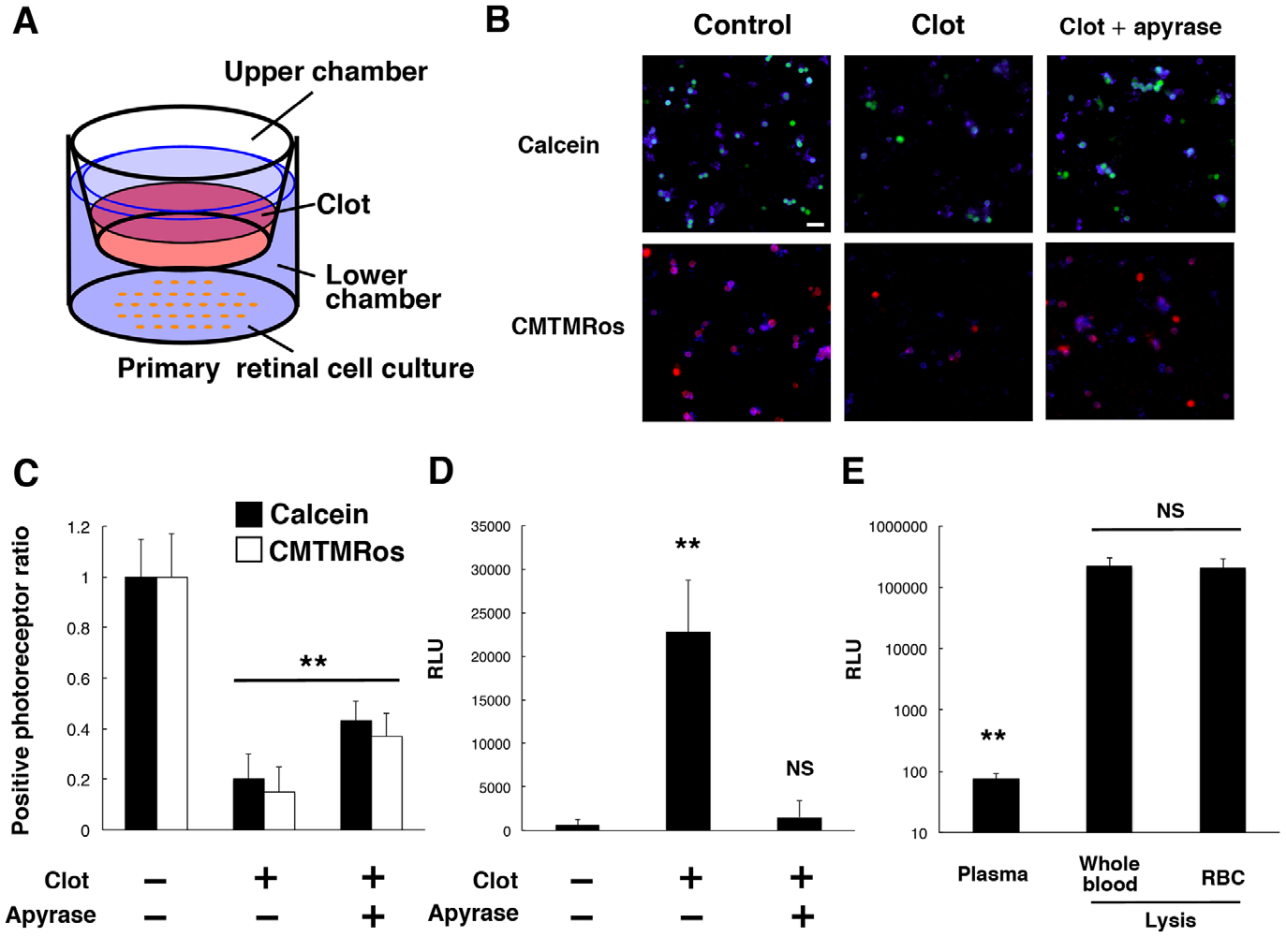
Photoreceptor cell apoptosis in primary retinal cell cultures with blood clots. (A) Schematic image of the double chamber co-culture system of primary retinal cells and blood clots. (B) and (C) The viability of primary retinal cells in the lower chamber was accessed by calcein AM or MitoTracker CMTMRos after 24 h of culture with addition of a clot in the upper chamber (calcein in green, CMTMRos in red, recoverin in blue). The frequency of calcein^+^ or CMTMRos^+^ photoreceptors significantly decreased after incubation with clots. Apyrase treatment significantly rescued photoreceptors. (D**)** The ATP levels of culture medium in the lower chamber were significantly increased by clot exposure, and reversed by apyrase treatment. (E**)** The ATP levels in plasma and blood. *n = *10 per group; **P<0.01. Scale bar: 20 µm.

To identify potential neurotoxic factors released from the extravascular blood clots, we prepared mouse primary retinal cells in double chamber slides. Primary mouse retinal cell cultures were prepared as previously described [22] [48] in the lower chamber, while mouse blood was added to the upper chamber on membranes with micropores (Figure 3A). In this double chamber system, released soluble factors can easily diffuse between two chambers, while the blood clot itself remains in the upper chamber. Primary retinal cell cultures were incubated for 24 h with or without addition of a clot to the upper chamber. The cultures were also examined in the absence or presence of 10 U/ml apyrase, a soluble ATP-degrading enzyme (ecto-ATPase) in the lower chamber. At 24 h of incubation, the clot showed a significant reduction in live photoreceptor cells (Figure 3, B and C), which were identified by two fluorescent live cell sensors, calcein AM and MitoTracker CMTMRos, which only label intact, non-apoptotic cells, and metabolically active cells, respectively. Of note, addition of apyrase, an ecto-ATPase, rescued photoreceptors from blood toxicity (Figure 3, B and C), suggesting that ATP released from clots was a critical factor in reducing photoreceptor cell survival. To confirm the ATP release from the clot, ATP levels in the lower chamber were analyzed by luciferase assay. The ATP levels were markedly elevated in the culture medium in the lower chamber after the addition of a clot in the upper chamber (Figure 3D). Apyrase treatment efficiently reversed the increase in ATP levels in the lower chamber (Figure 3D), demonstrating the contribution of extracellular ATP to the clot-induced toxicity consistent with the results of the cell viability assay. To clarify the source of extracellular ATP, we examined the ATP concentrations of mouse plasma. Only a small amount of ATP was detected in collected plasma, suggesting that the ATP levels in plasma were minimal under physiological conditions. However, massive ATP release was observed after hemolysis in whole blood (Figure 3E). In addition, ATP levels did not differ significantly between whole blood and isolated erythrocytes after hemolysis (Figure 3E), indicating that erythrocytes could be a dominant source of extracellular ATP from clots. Taken together, these results suggest that released extracellular ATP may be a potential neurotoxic factor in neovascular AMD with subretinal hemorrhage.

### Extracellular ATP Induces Photoreceptor Cell Apoptosis *in vitro* and *in vivo*

**Figure 4:**
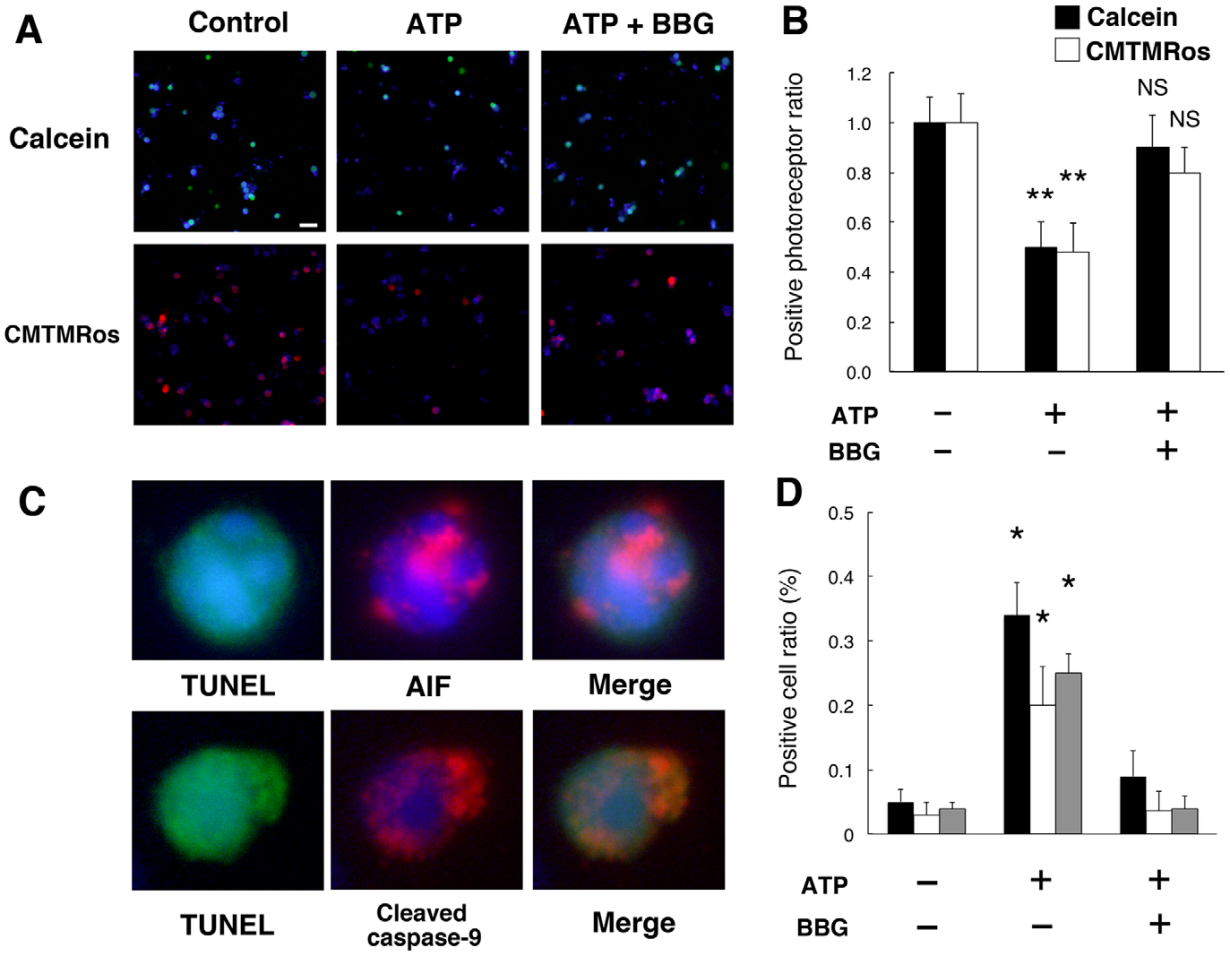
Photoreceptor cell apoptosis by ATP administration *in vitro*. (A) and (B) Calcein^+^ or CMTMRos^+^ photoreceptors were reduced by 24 h incubation of 1 mM ATP (calcein in green, CMTMRos in red, recoverin in blue). BBG notably attenuated the decline of viable photoreceptors. (C**)** and (D) Representative images of immuno-cytochemistry of AIF (top panels) and cleaved caspase-9 (bottom panels) in TUNEL^+^ cells in primary retinal cell cultures (TUNEL in green, AIF or cleaved caspase-9 in red, Hoechst 33342 in blue) and the quantifications. *n = *10 per group; *P<0.05. **P<0.01. Scale bar: 20 µm.

To obtain the mechanistic insights into the contribution of ATP/P2RX7 to photoreceptor death, we performed ATP administrations in primary culture. The addition of exogenous ATP significantly reduced viable, calcein^+^ or CMTMRos^+^ photoreceptor cells, while BBG reversed the decline in live photoreceptor cells (Figure 4, A and B). Furthermore, ATP administration induced TUNEL-detectable degradation of DNA with signs of the mitochondrial apoptotic pathway, namely AIF translocation to the nucleus and caspase-9 cleavage, whereas BBG treatment significantly inhibited those effects (Figure 4, C and D). These results indicate that extracellular ATP can trigger photoreceptor cell apoptosis via P2RX7-dependent machinery.

**Figure 5:**
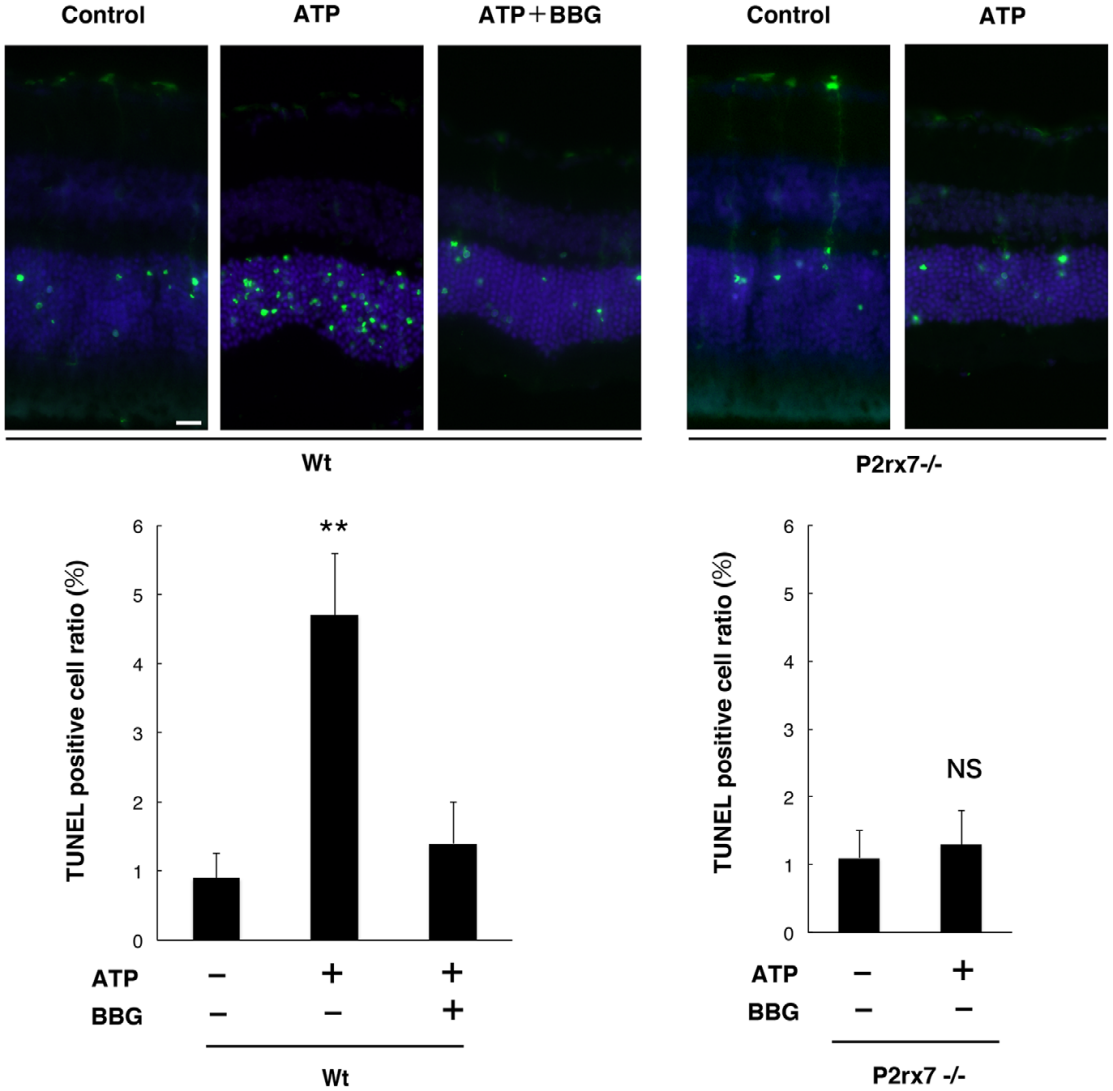
Photoreceptor cell apoptosis by subretinal injection of ATP. Exogenous ATP was injected into the subretinal space of Wt or P2rx7^−/−^ mice. TUNEL-positive apoptotic cells developed in the ONL 24 h after the subretinal injection of 1 mM ATP (TUNEL in green and Hoechst 33342 in blue). *n = *6 per group; **P<0.01. Scale bar: 20 µm.

Next, we measured retinal cell apoptosis in the presence or absence of subretinal administration of exogenous ATP to determine whether the increase of subretinal ATP could trigger photoreceptor cell death *in vivo*. Exogenous ATP was mixed with sodium hyaluronate and injected into the subretinal space of C57BL/6 mice. We confirmed that the height of retinal detachment in the presence of the ATP mixture was not significantly different from that in the absence of ATP mixture (Supporting Information S3). Subretinal injections without ATP induced only limited TUNEL-positive apoptotic events in the ONL after 24 h of injections (Figure 5), whereas TUNEL-detectable DNA degradation was significantly accelerated by the administration of 1 mM ATP in the subretinal space (Figure 5). The increased photoreceptor cell apoptosis was substantially reduced by administration of 50 µM BBG (Figure 5). Furthermore, the numbers of TUNEL^+^ photoreceptor cells were reduced in P2rx7^−/−^ mice, while there was massive loss of photoreceptors in wild-type (Wt) mice (Figure 5), indicating that the induction of apoptosis by P2RX7 activation was truly specific. Thus, ATP administration substantially mimicked the effects of ATP release from clots. Taken together, these results showed that excessive ATP facilitates photoreceptor cell apoptosis via P2RX7 activation *in vitro* and *in vivo*, indicating a pathological contribution of extracellular ATP in subretinal hemorrhage.

### BBG Protects Photoreceptors in a Mouse Model of Subretinal Hemorrhage

**Figure 6:**
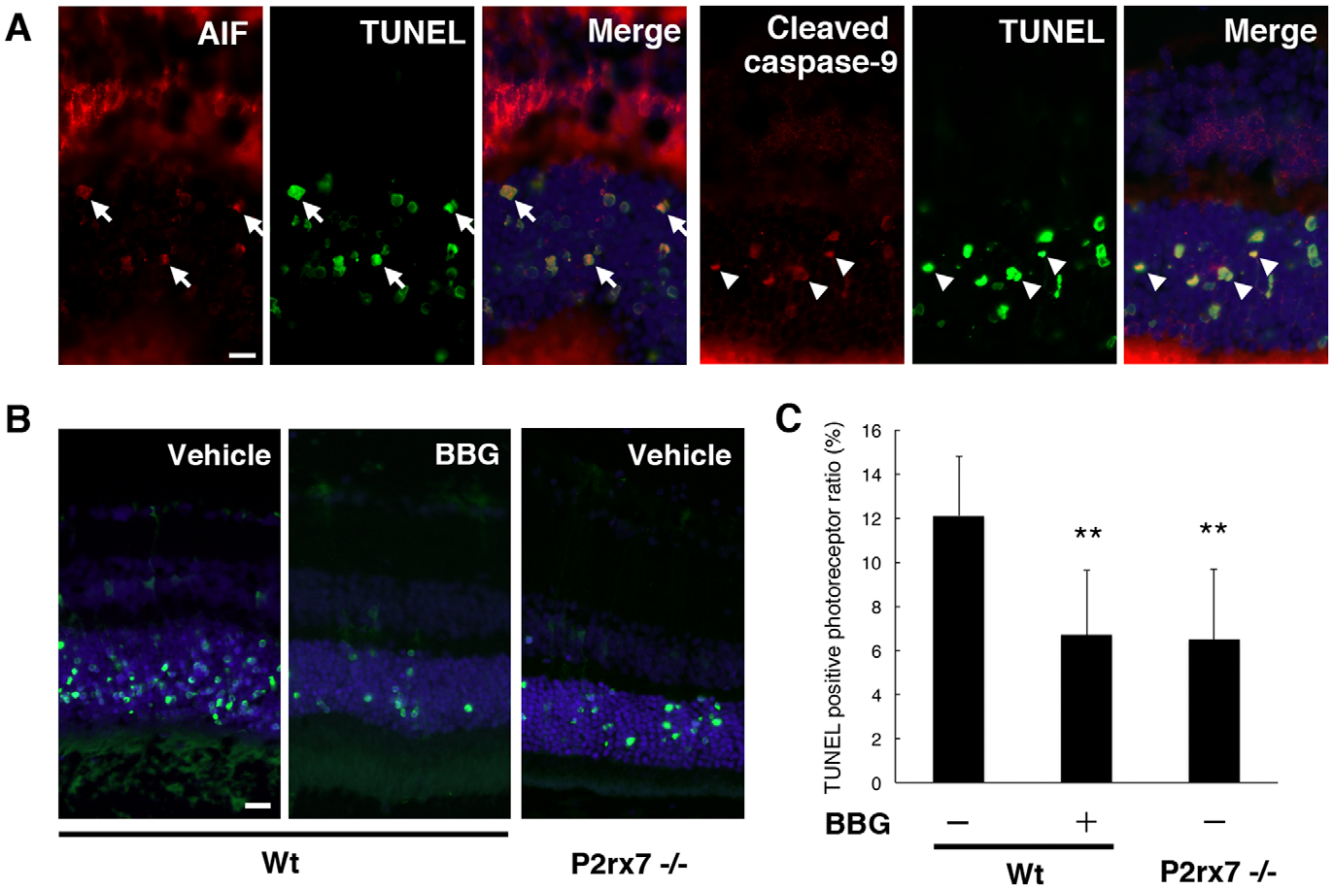
A P2RX7 antagonist prevents photoreceptor cell apoptosis in a mouse model of subretinal hemorrhage. (A) After the subretinal injection of autologous blood, photoreceptor cells underwent apoptotic cell death, and AIF-positive staining was observed in TUNEL^+^ photoreceptor nuclei (arrows; AIF in red, TUNEL in green, and Hoechst 33342 in blue). Caspase-9 cleavage was also detected among TUNEL^+^ nuclei (arrowheads; cleaved caspase-9 in red). Scale bar: 10 µm. (B) and (C) TUNEL^+^ apoptotic cells in the absence or presence of 50 µM BBG treatment after experimental subretinal hemorrhage in Wt or P2rx7^−/−^ mice. *n = *6 per group; **P<0.01. Scale bar: 20 µm.

The results obtained from clinical samples and the *in vitro* and *in vivo* experiments led us to investigate whether the pharmacological P2RX7 antagonist would have a beneficial effect on retinal damage in a more pathological setting–namely, an animal model of subretinal hemorrhage. Autologous blood was injected into the subretinal space alone or with a mixture of BBG (50 µM). Twenty-four hours after experimental subretinal hemorrhage, abundant TUNEL^+^ photoreceptor cells were detected in the ONL with AIF translocation to the nucleus and caspase-9 cleavage (Figure 6A) in line with our previous reports that photoreceptor cells undergo apoptosis via the mitochondrial pathway as a result of MOMP [22]. These effects were notably attenuated by local injection of BBG (Figure 6, B and C). Moreover, TUNEL-detectable photoreceptor cell apoptosis was reduced in P2rx7^−/−^ mice compared to Wt mice (Figure 6, B and C). Taken together, these results demonstrate that pharmacological inhibition of P2RX7 could results in neuroprotection of photoreceptors in cases of subretinal hemorrhage.

## Discussion

In the present work, we have shown that severe human hemorrhagic pathologies, in particular AMD, are accompanied by a dynamic increase in extracellular ATP, which worsens photoreceptor degeneration. ATP was released from extravascular blood in primary retinal cell cultures *in vitro* and in a model of subretinal hemorrhage *in vivo*, and triggered severe photoreceptor cell apoptosis via P2RX7 ligation. We also provided mechanistic insights into exogenous ATP-induced photoreceptor cell apoptosis via mitochondrial apoptotic pathways–namely, the activation of caspase-9 and the mitochondrio-nuclear translocation of AIF. To our knowledge, this is the first report on pathogenic increase of extracellular ATP in hemorrhagic disorders in the retina as well as in the CNS. Finally, we demonstrated that BBG, a pharmacological P2RX7 antagonist, could correct photoreceptor cell death in a rodent model of subretinal hemorrhage.

The clinical application of anti-VEGF antibodies has enabled the regulation of CNV progression in patients with AMD worldwide [6]. However, patients still tend to have poor prognosis in neovascular AMD after massive subretinal hemorrhage. AMD as well as intracerebral hemorrhage causes severe tissue damage, suggesting that extravascular blood may become highly toxic to surrounding cells through the release of toxins [49]. In the current study, based on *in vitro* and *in vivo* models of blood neurotoxicity, we proposed a novel pathological pathway by which extracellular ATP could mediate mitochondrial apoptotic signaling. Moreover, we found that extracellular ATP levels can substantially increase in the presence of subretinal hemorrhage. Several potential mechanisms may account for the increase in extracellular ATP in hemophagic disorders. Acute cell lysis could be a source of ATP, in line with previous observations that ATP is released by acute stresses such as ischemia [55], hypotony [56], or oxygen/glucose deprivation [57]. In the present study, we observed that some erythrocytes underwent hemolysis under the retina and that blood clot exposure caused significant elevation of extracellular ATP *in vitro*. Hence, hemolysis could be a part of the cause of the ATP increase in subretinal hemorrhage. Moreover, the massive ATP release may be caused by usurpation of the physiological mechanism of release. Indeed, erythrocytes are sensitive to low oxygen supply and upregulate ATP efflux to modulate vascular tone via P2Y receptors on the endothelium [58]. These effluxes of ATP from erythrocytes may account for the findings that there were sustained elevation of ATP levels in the vitreous samples from AMD but not in those from MH or ERM.

Our results demonstrated that subretinal hemorrhage induced massive photoreceptor cell apoptosis, while the level of apoptosis was limited in cases of vitreous hemorrhage. The finding that vitreous hemorrhage induced minimal retinal cell apoptosis could be explained as follows. First, ATP concentrations around photoreceptors could be much higher in conditions in which blood clots are tightly packed in the subretinal space, while ATP can rapidly disperse into the vitreous space in cases of vitreous hemorrhage. In addition, subretinal injections of toxic agents have a greater risk of toxicity than vitreous injections [59]–[60]. The difference in the effect between intravitreous and subretinal injections was found to be more than 40-fold. This might be due to the presence of internal limiting membrane (ILM) and Müller glial cells that separate the vitreous cavity and neural retina, or the various cellular sensitivities to the specific stimulant. Another plausible explanation for the various sensitivities to extracellular ATP is localization of ecto-nucleoside triphosphate diphosphohydrolases (NTPDase) in the retina. NTPDase 1 and NTPDase 2 are mainly located in the ganglion cell layer (GCL), inner plexiform layer (IPL), and outer plexiform layer (OPL), indicating synaptic localization. In the OPL, NTPDases mainly localize in postsynaptic processes but not photoreceptor terminals [61]. In subretinal hemorrhage, ATP released from blood might be degraded by NTPDase during its diffusion through those synaptic layers, while photoreceptors could be exposed to ATP before its degradation. Hence, photoreceptors rather than ganglion cells and other inner retinal cells may tend to undergo apoptosis. Also in the case of vitreous hemorrhage, ATP might be degraded during diffusion through NTPDase-abundant inner retinal layers, which could account for why ATP had less toxic effect in vitreous hemorrhage than in subretinal hemorrhage.

Our present results also showed that subretinal injections of ATP markedly accelerated photoreceptor cell apoptosis, whereas other cells in the retina underwent limited TUNEL-detectable apoptosis. However, ganglion cells are reported to undergo cell death in response to the vitreous injection of BzATP [62]. A possible explanation for this discrepancy is that the local ATP concentration might not be sufficient to induce cell death in the inner retina. In our experiment, the highest concentration of ATP is assumed to be localized around photoreceptor cells. In addition, the retinal pigment epithelium (RPE) may have played roles in the observed photoreceptor degeneration. RPE cells also express P2RX7 [63] and may undergo cell death by subretinal hemorrhage. RPE degeneration could further worsen the photoreceptor degeneration, because RPE provides neurotrophic factors essential for photoreceptor survival. Further investigations will be needed to elucidate the role of RPE and extracellular ATP in retinal degeneration.

BBG, a selective P2RX7 antagonist, has been shown to be a potential therapeutic agent in a mouse model of spinal cord injury [40] [64]. Hence, BBG administration may have a neuroprotective effect in neurodegenerative diseases potentially linked to excessive extracellular ATP. In this context, it is worth noting that BBG is a clinically approved adjuvant for the surgical procedure of chromovitrectomy. We have introduced BBG as a safe staining dye for chromovitrectomy and an increasing number of reports support the safety and efficacy of BBG [44] [65]–[68]. Taken together, these findings demonstrate that BBG is biocompatible with ocular tissues, including neurosensory retina. Our results further encourage the potential application of BBG as a neuroprotective agent in degenerative disorders, especially in ATP-releasing subretinal hemorrhage. Furthermore, we have also reported that dying cells could release their own intracellular ATP into the extracellular space, resulting in a positive feed-forward loop that worsens the surrounding tissue damage [37]. Our current study further suggests that similar severe neurodegenerative pathologies, such as subarachnoid hemorrhage or intracerebral hemorrhage, could be linked to important elevations of extracellular ATP, accelerating neuronal cell death and irreversible tissue damage. P2RX7 antagonists including BBG may have a neuroprotective therapeutic effect in retinal diseases as well as in CNS diseases with excessive extracellular ATP.

## Supporting Information

Supporting Information S1 Genotyping of the rd8 mutation by DNA sequencing in P2rx7^−/−^ mice. Recently, the rd8 mutation has been reported to be widely inherited across strains supplied by common commercial vendors and ES cell lines [45]–[46]. To confirm that P2rx7^−/−^ mice do not carry the rd8 mutation, we performed genotyping of the Crb1*^rd8^* allele carrying a single base pair deletion in exon 9 of the Crb1 gene. PCR amplification using flanking primers (5′-GCC CCT GTT TGC ATG GAG GAA ACT-3′ and 5′- GCC CCA TTT GCA CAC TGA TGA C-3′) and subsequent DNA sequencing demonstrated that the genotype of P2rx7^−/−^ mice is the wild type allele (*n* = 6). Ocular fundus examination also confirmed that P2rx7^−/−^ mice have no phenotype of retinal degeneration, namely drusen-like lesions, which are found in mice carrying the rd8 mutation [46].
(PDF)

Supporting Information S2 Characteristics and clinical data in patients with vitreous hemorrhage due to AMD. About 2,000 AMD patients are referred to our Vitreoretinal Center every year. Patients undergo ophthalmologic examinations, including visual acuity testing with standardized refraction using decimal charts and slit-lamp biomicroscopy. Decimal fractions of visual acuity were converted to the logarithm of the minimal angle of resolution (LogMAR) according to previous reports [69]–[70]. The mean preoperative visual acuity in AMD with VH was 0.008/2.10 (decimal/LogMAR), ranging from light perception to 0.03. At the preoperative examinations, the ocular fundi were almost invisible due to vitreous hemorrhage in AMD patients who underwent vitrectomy. After surgical removal of the vitreous opacity, the area of subretinal hemorrhage was confirmed by a postoperative review of the video records. A large area of subretinal hemorrhage ranging 4 to 30 disc diameters that involves the macula was observed in the fundus of those patients.
(TIF)

Supporting Information S3 The height of retinal detachment by injections of sodium hyaluronate, sodium hyaluronate with a mixture of ATP, and autologous blood. The height of retinal detachment was measured as the length between the photoreceptor outer segment and RPE in cryosections. The mean lengths in the three groups were not significantly different.
(TIF)
