# Supplementary material for: Dynamic Increase in Extracellular ATP Accelerates Photoreceptor Cell Apoptosis via Ligation of P2RX7 in Subretinal Hemorrhage
Source: PLoS One. 2013 Jan 8;8(1):e53338. doi: 10.1371/journal.pone.0053338 (PMC3540091; doi:10.1371/journal.pone.0053338)
Supplement: Supporting Information S1 — Genotyping of the rd8 mutation by DNA sequencing in P2rx7−/− mice. Recently, the rd8 mutation has been reported to be widely inherited across strains supplied by common commercial vendors and ES cell lines [45]–[46]. To confirm that P2rx7−/− mice do not carry the rd8 mutation, we performed genotyping of the Crb1rd8 allele carrying a single base pair deletion in exon 9 of the Crb1 gene. PCR amplification using flanking primers (5′-GCC CCT GTT TGC ATG GAG GAA ACT-3′ and 5′- GCC CCA TTT GCA CAC TGA TGA C-3′) and subsequent DNA sequencing demonstrated that the genotype of P2rx7−/− mice is the wild type allele (n = 6). Ocular fundus examination also confirmed that P2rx7−/− mice have no phenotype of retinal degeneration, namely drusen-like lesions, which are found in mice carrying the rd8 mutation [46]. (PDF) [file pone.0053338.s001.pdf]

**Supplemental Information S1**

**Genotyping of the rd8 mutation by DNA sequencing in P2rx7$^{-/-}$ mice.**

Recently, the rd8 mutation has been reported to be widely inherited across strains supplied by common commercial vendors and ES cell lines [45] [46]. To confirm that P2rx7$^{-/-}$ mice do not carry the rd8 mutation, we performed genotyping of the Crb1$^{rd8}$ allele carrying a single base pair deletion in exon 9 of the Crb1 gene. PCR amplification using flanking primers (5'-GCC CCT GTT TGC ATG GAG GAA ACT-3' and 5'-GCC CCA TTT GCA CAC TGA TGA C-3') and subsequent DNA sequencing demonstrated that the genotype of P2rx7$^{-/-}$ mice is the wild type allele ($n$ = 6). Ocular fundus examination also confirmed that P2rx7$^{-/-}$ mice have no phenotype of retinal degeneration, namely drusen-like lesions, which are found in mice carrying the rd8 mutation [46].
