# Supplementary figures and images for: Dynamic Increase in Extracellular ATP Accelerates Photoreceptor Cell Apoptosis via Ligation of P2RX7 in Subretinal Hemorrhage
Source: PLoS One. 2013 Jan 8;8(1):e53338. doi: 10.1371/journal.pone.0053338 (PMC3540091; doi:10.1371/journal.pone.0053338)

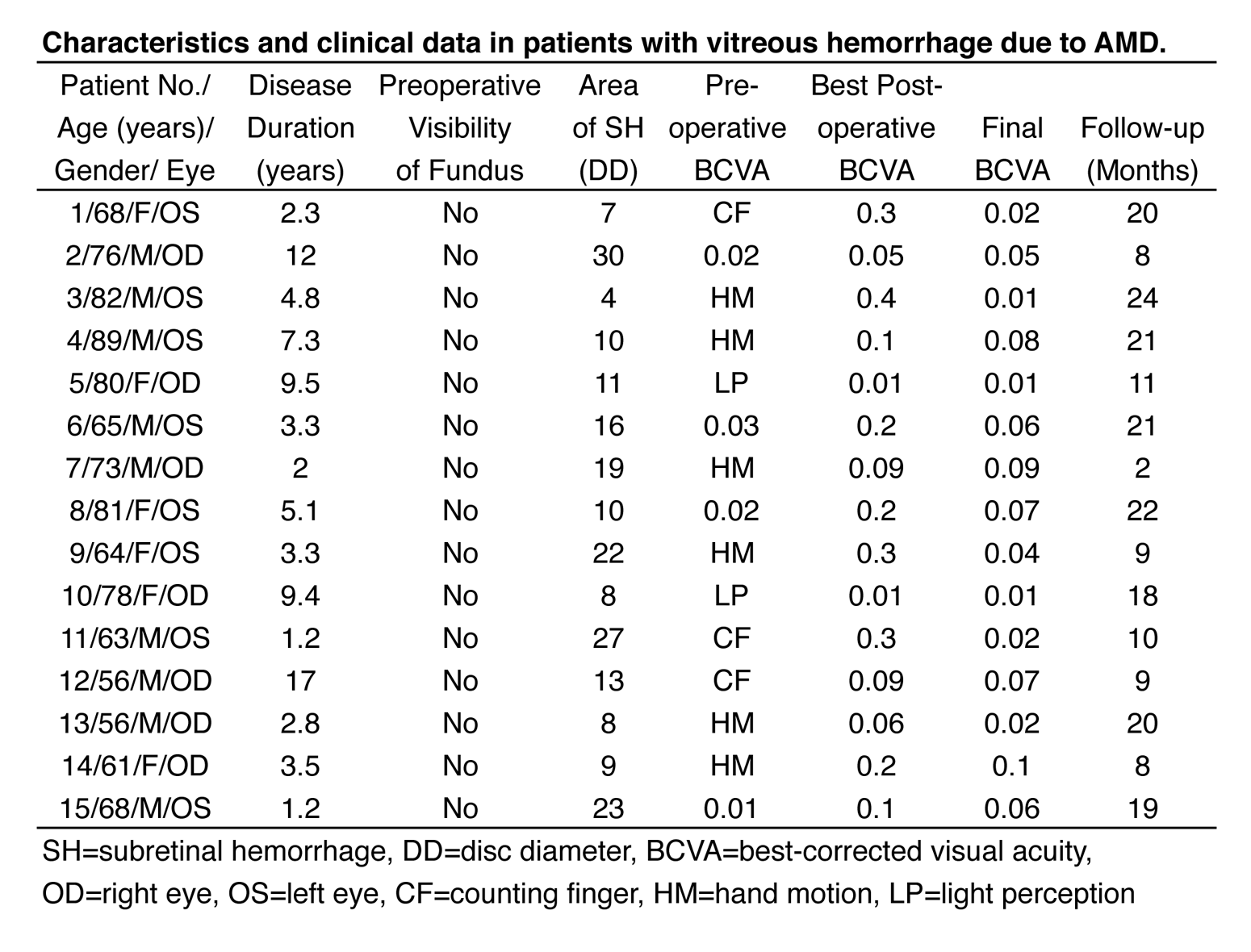

Supplement: Supporting Information S2 — Characteristics and clinical data in patients with vitreous hemorrhage due to AMD. About 2,000 AMD patients are referred to our Vitreoretinal Center every year. Patients undergo ophthalmologic examinations, including visual acuity testing with standardized refraction using decimal charts and slit-lamp biomicroscopy. Decimal fractions of visual acuity were converted to the logarithm of the minimal angle of resolution (LogMAR) according to previous reports [69]–[70]. The mean preoperative visual acuity in AMD with VH was 0.008/2.10 (decimal/LogMAR), ranging from light perception to 0.03. At the preoperative examinations, the ocular fundi were almost invisible due to vitreous hemorrhage in AMD patients who underwent vitrectomy. After surgical removal of the vitreous opacity, the area of subretinal hemorrhage was confirmed by a postoperative review of the video records. A large area of subretinal hemorrhage ranging 4 to 30 disc diameters that involves the macula was observed in the fundus of those patients. (TIF) [file pone.0053338.s002.tif]

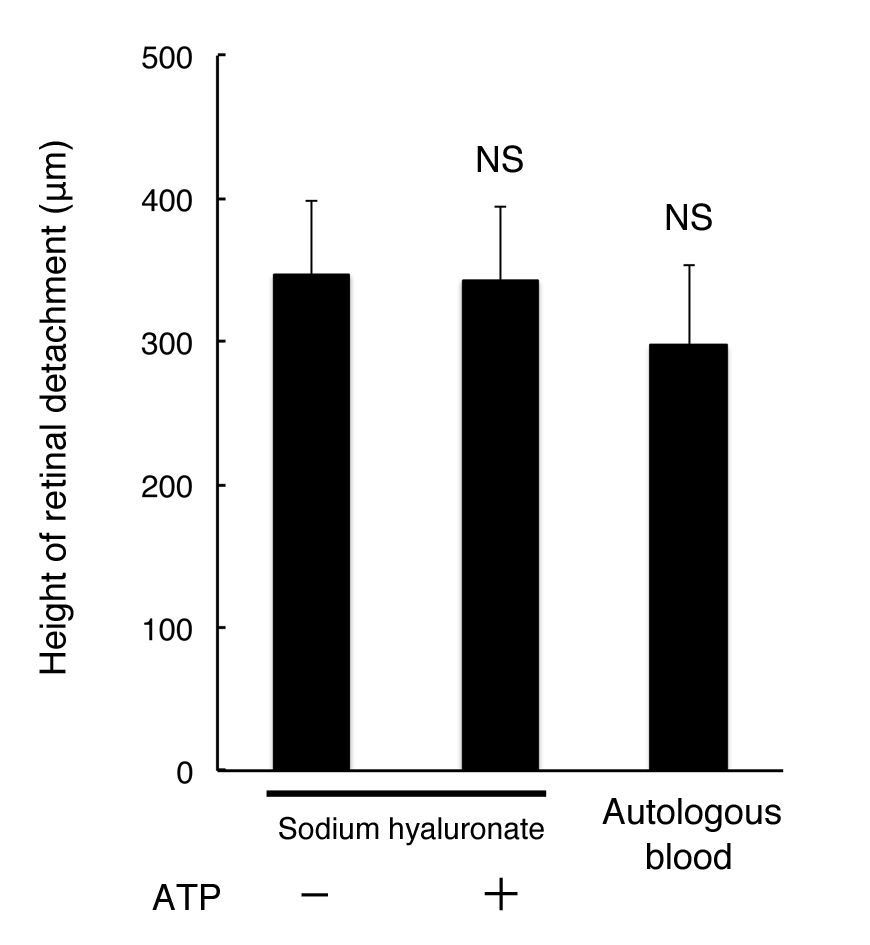

Supplement: Supporting Information S3 — The height of retinal detachment by injections of sodium hyaluronate, sodium hyaluronate with a mixture of ATP, and autologous blood. The height of retinal detachment was measured as the length between the photoreceptor outer segment and RPE in cryosections. The mean lengths in the three groups were not significantly different. (TIF) [file pone.0053338.s003.tif]
